# SARS-CoV-2 Whole-Genome Sequencing by Ion S5 Technology—Challenges, Protocol Optimization and Success Rates for Different Strains

**DOI:** 10.3390/v14061230

**Published:** 2022-06-06

**Authors:** Maria Szargut, Sandra Cytacka, Karol Serwin, Anna Urbańska, Romain Gastineau, Miłosz Parczewski, Andrzej Ossowski

**Affiliations:** 1 Department of Forensic Medicine, Pomeranian Medical University in Szczecin, 70-111 Szczecin, Poland; sandra.cytacka@pum.edu.pl (S.C.); andrzej.ossowski@pum.edu.pl (A.O.); 2 Department of Infectious, Tropical Diseases and Immune Deficiency, Pomeranian Medical University in Szczecin, 71-455 Szczecin, Poland; karol.serwin@pum.edu.pl (K.S.); anna.urbanska@pum.edu.pl (A.U.); milosz.parczewski@pum.edu.pl (M.P.); 3 Institute of Marine and Environmental Sciences, University of Szczecin, 70-383 Szczecin, Poland; romain.gastineau@usz.edu.pl

**Keywords:** SARS-CoV-2, whole-genome sequencing, variants of concern, NGS, COVID-19, molecular epidemiology, library preparation

## Abstract

The COVID-19 pandemic demonstrated how rapidly various molecular methods can be adapted for a Public Health Emergency. Whether a need arises for whole-genome studies (next-generation sequencing), fast and high-throughput diagnostics (reverse-transcription real-time PCR) or global immunization (construction of mRNA or viral vector vaccines), the scientific community has been able to answer all these calls. In this study, we aimed at the assessment of effectiveness of the commercially available solution for full-genome SARS-CoV-2 sequencing (AmpliSeq™ SARS-CoV-2 Research Panel and Ion AmpliSeq™ Library Kit Plus, Thermo Fisher Scientific). The study is based on 634 samples obtained from patients from Poland, with varying viral load, assigned to a number of lineages. Here, we also present the results of protocol modifications implemented to obtain high-quality genomic data. We found that a modified library preparation protocol required less viral RNA input in order to obtain the optimal library quantity. Concurrently, neither concentration of cDNA nor reamplification of libraries from low-template samples improved the results of sequencing. On the basis of the amplicon success rates, we propose one amplicon to be redesigned, namely, the r1_1.15.1421280, for which less than 50 reads were produced by 44% of samples. Additionally, we found several mutations within different SARS-CoV-2 lineages that cause the neighboring amplicons to underperform. Therefore, due to constant SARS-CoV-2 evolution, we support the idea of conducting ongoing sequence-based surveillance studies to continuously validate commercially available RT-PCR and whole-genome sequencing solutions.

## 1. Introduction

The COVID-19 outbreak, characterized on the 11 March 2020 as a pandemic by the World Health Organization (WHO), demonstrated how robust, precise, and flexible molecular diagnostic methods are. Just a few days after the WHO’s director-general declared the epidemic a Public Health Emergency of International Concern (PHEIC) on 30 January 2020 [1], the Center for Disease Control and Prevention (CDC) submitted an application to the Agency of Food and Drug Administration (FDA) for an Emergency Use Authorization (EUA) license of a real-time PCR test that could detect two fragments of the N gene of SARS-CoV-2. The FDA granted the EUA the next day. CDC has made the test design public, thus allowing hundreds of laboratories worldwide to help prevent further viral spread [2]. As of February 2022, 248 molecular tests for detection of nucleic acids have been approved in the United States of America for testing against SARS-CoV-2 in certified laboratories or patient care settings. Additionally, 25 have been authorized as laboratory-developed tests (LDTs) for use in laboratories that meet requirements to perform high-complexity tests [3].

Initially, a limited number of SARS-CoV-2 genome sequences was sufficient for the construction of commonly used molecular tests—at the time of the publication of the CDC 2019-nCoV RT-PCR Diagnostic Panel, less than 500 full genome sequences were included in public databases [4]. However, to understand the virus’s transmission patterns, the need for whole-genome studies arose [5,6,7,8]. Moreover, as time progressed and the number of infected people grew, the virus mutated, as expected, with some strains prevailing within regions, countries, or even continents. The changed nucleotide sequence of the virus can impact the epidemiology of the outbreak, as well as the clinical picture and severity of COVID-19 [9]. On this account, the WHO established the definitions of a variant under monitoring (VUM), variant of interest (VOI) and variant of concern (VOC). When genetic changes are suspected to affect the virus characteristics in a way that may pose a future risk, but there is not enough data to confirm or reject this hypothesis yet, the variant is qualified as a VUM. Both VOI and VOC are characterized as an emerging risk to global public health, with VOC additionally having either increased transmissibility/causing detrimental change in COVID-19 epidemiology; having increased virulence/different clinical disease presentation; or causing a decrease in effectiveness of public health and social measures or available diagnostics, vaccines, or therapeutics. At the time of submission of this article, the WHO designated two VOIs: Lambda (on the 14 June 2021; C.37. according to Pango nomenclature) and Mu (30 August 2021; B.1.621), and five VOCs: Alpha and Beta (both: 18 December 2020; B.1.1.7 and B.1.351, respectively), Gamma (11 January 2021; P.1), Delta (11 May 2021–VOI since 4 April 2021; B.1.617.2), and Omicron (26 November 2021; B.1.1.529). At the same time, three VUMs are designated: B.1.1.318 (VUM since 2 June 2021), C.1.2 (1 September 2021), and B.1.640 (22 November 2021) [10].

The genetic changes that the virus undergoes can also affect the results of routine molecular tests performed to detect the presence of SARS-CoV-2 RNA in clinical samples. For this reason, the FDA has performed analyses of impact of widely spread mutations on several commercially available, real-time RT-PCR-based SARS-CoV-2 RNA detection kits. In case of three of them, the FDA stated that one of the targets has significantly reduced sensitivity due to certain mutations, including one of the mutations in the Alpha variants. The presence of those mutations, and in return failure in target amplification, do not pose a risk of falsely negative results as a multi target approach is applied within these tests [11]. Still, the most recent VOC—the Omicron variant—shows an unprecedented number of mutations compared to the SARS-CoV-2 Wuhan-Hu-1 sequence [12]. Overall, S-gene dropout is expected in two and N-gene drop out is expected in 26 FDA-approved tests when the Omicron variant is being tested; however, due to the multi-target approach employed within these tests, their sensitivity should not be impacted. Nevertheless, the FDA has recommended that two real-time RT-PCR tests (one single target and one multi target) should not be used due to their expected inability to detect the Omicron variant, and additionally one single target test has been modified specifically to address this issue and has been cleared for use [11]. This demonstrates the importance of conducting an ongoing whole-genome-sequencing-based epidemiologic surveillance throughout the pandemics, even after initial genetic pathogen identification is completed.

In this study, we aimed to assess the effectiveness of the AmpliSeq™ SARS-CoV-2 Research Panel and Ion AmpliSeq™ Library Kit Plus (Thermo Fisher Scientific) for sequencing of whole SARS-CoV-2 genomes in samples with varying viral load that are assigned to a number of lineages, including two VOCs: Alpha and Delta. Here, we also show the results of protocol modifications implemented to obtain high-quality genomic data.

## 2. Materials and Methods

For the research material, we have chosen nasal/nasopharyngeal swabs collected from 665 individuals from Poland who were diagnosed with COVID-19 on the basis of a reverse-transcription real-time PCR test specific to SARS-CoV-2 genome fragments.

RNA was extracted from samples with the use of a MagMAX™ Viral/Pathogen Nucleic Acid Isolation Kit and a KingFisher™ Flex Purification System (both: Thermo Fisher Scientific™, TFS). Isolates were then subjected to a confirmative real-time PCR test (TaqPath™ COVID-19 CE-IVD RT-PCR Kit, TFS) to assess the viral copy number in each isolate. cDNA was synthesized from each isolate with the use of SuperScript™ VILO™ Master Mix (TFS) as per the user manual. For some of the samples that did not contain the optimal RNA quantity, cDNA concentration was performed: 20 μL of cDNA synthesis product was centrifuged in Concentrator plus (Eppendorf) for 15 min at 45 °C (V-AQ mode), after which, sterile water was added for a final volume of 10 μL. According to the cycle in which the N-gene fluorescence signal exceeded 10,000 units (C_T_), samples were subjected either to a normalization or no-normalization protocol, and target regions were amplified with an Ion AmpliSeq™ SARS-CoV-2 Research Panel and an Ion AmpliSeq™ Library Kit Plus (both TFS), with two primer pools protocol. For the normalization protocol (original protocol suggested by the user manual), used for samples from runs R1 and R2, RNA extracts were divided into three groups on the basis of the C_T_ value: ≤22-samples were normalized to 20,000 viral copy number/reaction and amplified for 17 cycles, 23–25-samples were normalized to 2500 viral copy number/reaction and amplified for 20 cycles, and 26–31-samples were normalized to 78 viral copy number/reaction and amplified for 25 cycles. In the no-normalization protocol (modified protocol suggested by the manufacturing company in verbal communication), used for samples from runs R3–R8, RNA extracts were divided into four groups on the basis of the C_T_ value: 12–17-samples were amplified for 13 cycles, 18–22-samples were amplified for 18 cycles, 23–27-samples were amplified for 23 cycles, and > 27-samples were concentrated for 15 min in 45 °C, and afterwards, water was added for a final volume of 10 μL and samples were amplified for 27 cycles. Further steps of library preparation were performed with Ion AmpliSeq™ Library Kit Plus and Ion Xpress™ Barcode Adapters 1–96 Kit (both TFS) according to the manufacturer’s instructions. Barcoded libraries were purified with Agencourt™ AMPure™ XP Reagent (Beckman Coulter), eluted in 50 μL of TE buffer, and quantified by Ion Library TaqMan™ Quantitation Kit (TFS). Depending on the batch, selected libraries that did not meet the target concentration (70 pM for the selected chip type) were subjected to 5 cycles of amplification with 1X Library Amp Mix and 25X Library Amp Primers (contents of AmpliSeq™ Library Kit Plus) and purified again with Agencourt™ AMPure™ XP Reagent (this part of the procedure is described in the text as library reamplification). The concentration of double-stranded DNA in reamplified libraries was assessed by fluorometry (Qubit™ dsDNA HS Assay Kit, Qubit™ Flex Fluorometer, both TFS), with target concentration set at 18 ng/mL. All libraries were then diluted in TE buffer to their target concentration; subjected to templating in four runs, two chips each, performed by the Ion Chef™ Instrument; and shortly afterwards subjected to next-generation sequencing on the Ion GeneStudio™ S5 System, all with the use of Ion 540™ Kit-Chef and Ion 540™ Chip Kit (all above TFS). 

Sequencing results were analyzed in Torrent Suite™ Software with SARS-CoV-2 plugins: variantCaller, SARS_CoV_2_coverageAnalysis, and IRMAreport (all TFS) with standard configuration. FASTA files of sequences coming from libraries with optimal concentration were used for lineage assignment by Pangolin COVID-19 Lineage Assigner (https://pangolin.cog-uk.io/, accessed on 28 February 2022), and sequences that passed the internal PANGOLIN QC were further analyzed. BAM files were uploaded into the Integrative Genomic Viewer to help visualize the data. In the process of GISAID submission, 59 out of 414 sequences were handed back for revision due to the occurrence of frameshifts. Out of those, according to the IGV view, 53 were caused by either extremely low numbers of reads or no reads within different fragments of viral genomes, falsely interpreted by the IRMA plugin as deletions. For the manual sequence alterations Sequencher 5.4.6 was used. Sequences in which incorrect deletion calls occurred towards the first or last positions in reference to the Wuhan-Hu-1 were manually trimmed; when the frameshift occurred between correctly covered positions, incorrect deletion calls were manually filled with “N”. After the manual sequence alterations, sequences were re-uploaded to GISAID. When statistical calculations were performed, Statistica 13 was used.

## 3. Results

Out of 151,539,288 addressable wells within each 540 Chip, between 76,281,136 (50.3%) and 97,476,479 (64.3%) were live wells with library template that were not filtered out due to low-quality, polyclonal sequences or adapter dimers (for details, see Figure S1). Within all aligned base calls, at least 8.6 G perfectly aligned bases (with no measurable error) and at least 13.4 G bases with an error rate of ≤2% (AQ17) were called in each sequencing run (Table S1).

### 3.1. Genome Coverage

For all 634 samples tested, the mean target base coverage at 1× 20×, 100×, and 500×, one of the parameters indicating general genome quality were 96.70%, 91.48%, 88.47%, and 84.71%, respectively. The values obtained for the original, normalization protocol (96.18%, 85.33%, 78.62%, and 71.74%, respectively) were overall lower than those obtained for samples that had undergone the modified, no-normalization protocol (96.87%, 93.54%, 91.76%, and 89.05%, respectively) (for details, see Table S4).

To assess the correlation between the genome coverage and sample and library parameters, as well as some quality metrics, Spearman’s rank correlation coefficient (ρ) was calculated for target base coverage at 100×. A significant correlation (*p* > 0.05) was found for target base coverage at 100× and C_T_ value (moderate negative correlation, ρ = −0.42), and meeting the optimal library concentration (moderate positive correlation, ρ = 0.54). Within the sequencing metrics, a significant correlation (*p* > 0.05) was found for target base coverage at 100×, as well as total number of reads obtained for the sample (moderate positive correlation, ρ = 0.54), on target reads (moderate positive correlation, ρ = 0.61), and mean depth (moderate positive correlation, ρ = 0.54). Additionally, the correlation between sequencing metrics and C_T_ value was measured. A significant (*p* > 0.05) correlation was found for C_T_ value and on target reads (moderate negative correlation, ρ = −0.51) and uniformity (moderate negative correlation, ρ = −0.40). These results demonstrate that the quality of data obtained from low-template material is generally lower. Results of all correlation testing are included in Table S2.

### 3.2. Protocol Optimization and Modification of Low-Template Sample Library Preparation

**Figure 1:**
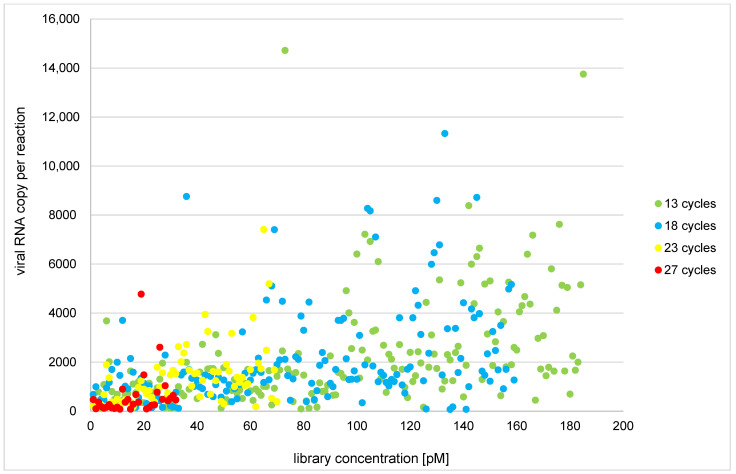
Correlation between initial RNA input and library construction with regard to the number of amplification cycles (no-normalization group and no library reamplification).

Out of 634 analyzed samples, 565 RNA isolates (89.3%) had a viral copy number optimal for library construction, and 561 (88.5%) yielded optimal library concentration (≥70 pM). Conversely, for samples with optimal RNA input, only 51.3% (R1) and 57.5% (R2) of samples amplified with the original normalization protocol produced an optimal library concentration. The modified, no-normalization protocol showed a 100% amplification success rate. The correlation between initial viral RNA input and library quantity within the no-normalization group for samples that did not undergo library reamplification is shown in Figure 1.

The no-normalization protocol produced optimal library concentration for samples with higher C_T_ values (the number of cycles in which the N-gene fluorescence signal exceeded 10,000 units), and thus lower initial RNA input; the mean N gene C_T_ for samples that generated libraries ≥ 70 pM was 21.5 in the no-normalization group and 18.7 for the normalization group (Table 1).

Overall, 69 isolates contained less viral RNA than the manufacturer recommends for library construction. A total of 59 of those isolates were subjected to cDNA concentration to utilize all cDNA that was synthesized from the sample, instead of just 5 μL that is used for standard cDNA synthesis and library preparation. As the mean viral RNA copy per standard reaction was similar between both groups of samples: 99.3 within the 59 isolates later subjected to RNA concentration and 104.0 for the remaining 10, the concentrated isolates contained about 2× more RNA than those that were not subjected to concentration, which would mean the optimal copy number per reaction should have been achieved for those samples. To assess the correlation between the concentration and some quality metrics, Spearman’s rank correlation coefficient (ρ) was calculated, with the process of RNA concentration being given a value of 1 and no concentration the value of 0. The ρ was −0.08, −0.03, −0.04, and −0.07 for number of reads per sample, target reads, mean sequencing depth, and uniformity, respectively (*p* > 0.05 for all four calculations). Results of all correlation testing are included in Table S2. The correlation coefficient indicates that neither library concentration nor sequencing quality metrics differed significantly between the two low-template groups (Table 2), and thus the concentration of cDNA did not improve the results of sequencing low-template samples.

### 3.3. Library Reamplification

From 73 libraries that did not meet the quantity requirement, 67 were chosen for library reamplification; out of those, 58 (86.6%) yielded optimal reamplified library concentration (≥18 ng/mL) (see Table 2). However, when sequenced, the reamplified libraries resulted in only 80.0% on target reads, with mean depth of 723.8 and 32.84% uniformity, with very little difference from when only reamplified libraries that met the concentration requirement were analyzed (79.81%, 801.0, and 35.36%, respectively). To assess the correlation between meeting the threshold of reamplified library concentration and some quality metrics, ρ was calculated, with the reamplified library concentration ≥ 18 ng/mL being given a value of 1 and <18 ng/mL the value of 0. The ρ was 0.38, −0.12, 0.31, and 0.10 for number of reads per sample, target reads, mean sequencing depth, and uniformity, respectively (*p* > 0.05 for all four calculations). This indicated that even though some data can still be obtained from libraries low in DNA quantity, meeting the threshold set for reamplified libraries concentration does not assure sequencing quality in a manner that the initial library quantity does (Table 3).

As only low-quality samples generated insufficient library concentration in the no-normalization group, libraries that were reamplified originated exclusively from samples with low initial RNA input in said group (mean C_T_ value: 31.0, meaning the viral RNA copy number per reaction more than two times less than recommended by the manufacturer). In the normalization group, the mean C_T_ value was 22.3 (Table 3). Therefore, the apparent superiority of the normalization protocol within the number of on target reads, mean depth, and uniformity obtained for the reamplified libraries seems to result from higher initial quality of the samples subjected to it, not the efficiency of the protocol itself.

### 3.4. Amplicon Success Rates

Out of 634 libraries subjected to IonTorrent sequencing, sequences of 633 whole/near-whole/partial genomes were obtained. Since 160 libraries (resulting in 159 sequences) were constructed with the use of the less efficient normalization protocol, all further calculations will be based on 414 samples that were subjected to the no-normalization library construction protocol and resulted in optimal library concentration (see Table 1, above).

Out of the 237 viral amplicons included in the Ion AmpliSeq™ SARS-CoV-2 Research Panel, for 236, at least 50 reads were obtained for 96% of samples. The one amplicon that consistently failed—r1_1.15.1421280—which covers positions 14,410–14,550 of the SARS-CoV-2 genome and lays within the ORF1ab, produced less than 50 reads for over 44% of samples (Table S3a). As the second to worst result was failure to obtain at least 50 reads for only 3.9% of samples, the need to redesign the primers for r1_1.15.1421280 amplicon is clearly visible. Since the panel was designed in a way that the amplicons overlapped, the sequence potentially lost because r1_1.15.1421280 failure was 43 bp long (14,472–14,515). Within all samples studied in the presented research, only one variant was found in that range (14476T>C, a missense variant leading to an amino acid substitution Tyr4738His; sample 366). To compare the number of reads obtained for each amplicon with the total number of reads for respective samples, mean percentage of amplicon reads was calculated (Table S3b). As some variation between amplicon success rates is expected, three categories of amplicon performance were set on the basis of the calculated mean percentage of share (100%/237 amplicons ≈ 0.422%): highly underperforming amplicons (mean percentage of amplicon’s reads within total reads per sample less than 5%; <0.021%), underperforming amplicons (more than 5% and less than 30%; >0.021% and <0.127%), and well-performing (more than 30%; >0.127%). Out of all 237 viral amplicons, 234 fell in the well-performing category, with just two underperforming amplicons (r1_1.4.295991-positions 3232–3453, and r1_1.23.127614–positions 21,757–21,973) and one highly underperforming amplicon (previously described r1_1.15.1421280). No statistically significant correlation between amplicon length and mean percentage of amplicon’s reads within total reads were found through Spearman’s R test (ρ = −0.086, *p* = 0.19; Table S2).

### 3.5. Performance through Strains

To assess the impact of newly occurring mutations (as of August 2021) on the panel performance, the samples were grouped into lineages, and mean percentage of amplicon’s reads within total reads per sample were calculated for each lineage. All amplicons that highly underperformed within studied lineages are shown in Table 4.

In almost every case when an amplicon was highly underperforming, a mutation was found either downstream (4–24 positions, mean 15) or upstream (0–19 positions, mean 7.7) of the amplicon. Moreover, every time the mutation was also found outside of the particular lineage, it caused the amplicon to underperform (5/19 cases) or, more frequently, highly underperform (14/19 cases). In one case, a heteroplasmic mutation was observed in a sample in the same position as one associated with amplicon failure within a different lineage (r1_1.26.1209362, lineage B.1.221–25906G>C; sample 100–25906G/C), but the heteroplasmy did not cause the amplicon to fail.

Since the study material was collected no later than August 2021, no sample including the VOC B.1.1.529 was included in the hereby presented analysis. To try and assess if the new, highly mutated B.1.1.529 variant would be prone to some amplification failure, five high-coverage sequences originating in Africa and described as Omicron variant were downloaded from the GISAID database (www.epicov.org, accessed on 28 February 2022): EPI_ISL_7834462 and EPI_ISL_9002859 (originating in Botswana), EPI_ISL_8065963 (Ghana), and two South African samples later withdrawn from the database (sequences included in Text S1). After comparing the B.1.1.529 sequences with the original reference, between 54 and 98 single-nucleotide changes were noted (N calls excluded). Out of those, 27 SNPs were shared between all five samples, and for those, the analysis of potential amplicon failure was performed. A total of 20 SNPs were located exclusively within ranges of amplicons, while 7 located up- or downstream of the amplicon range can be considered a potential source for amplicon underperformance (Table 5). Additionally, the multi nucleotide deletion at 28,362–28,370, which was present in four out of five sequences tested, known to cause the failure of amplification within several molecular tests, was included in the table.

Seeing how SNPs located several nucleotide positions up- or downstream of amplicon range could affect the amplicon’s performance, a similar analysis was performed for the underperforming and highly underperforming amplicons. At position 14,408 (2 nucleotide positions upstream of r1_1.15.1421280), a C > T SNP was found in 400 out of 414 samples. However, when mean percentage of reads within total reads per sample was calculated for each 14408C and 14408T variant samples, the values were equal for both sample groups (0.007%), and thus the significance of the 14408C>T SNP was rejected. In 288 samples, a multi nucleotide deletion starting at position 21,991 (18 nucleotide positions downstream of r1_1.23.127614) was found (21991delT, 21992delT, 21993delA) in a hotspot known for its presence in B.1.1.7 lineage (corresponding to Y144del). On the basis of an almost fivefold difference in mean percentage of amplicon reads within total reads (0.043% and 0.214% for samples with the deletion variant and 126 remaining, respectively), an assumption that the deletion has an impact on the overall SNP performance can be drawn. For the last underperforming amplicon (r1_1.4.295991–range 3232–3453), only one sample showed a mutation in relation to the reference sequence nearby the amplicon range (3478A>T). Even though the percentage of reads for this amplicon within total sample reads for the sample in question was more than five times lower than the mean value (0.026% vs. 0.116%), as this event occurred only in one sample, the amplicon’s underperformance was likely caused by another factor.

## 4. Discussion

The use of next-generation sequencing technology can be beneficial in several steps of an epidemic response [13], among them, pathogen identification [14], tracing the origins of the agent [15,16,17], and monitoring its spread [18,19,20,21,22,23] and evolution [17,24,25], as well as supporting development of diagnostic solutions and new therapeutic target discovery [26]. Even though there are different approaches to full-genome sequencing, the multiplex PCR amplicon method is currently one of the cheapest, while also proving to be superior to capture-based sequencing in the analysis of challenging samples [4]. For the purpose of the project founded by the Polish National Centre For Research and Development “Development of modern laboratory technologies, IT and bioinformatics dedicated to the diagnosis and prevention of SARS-CoV-2 infections” [27], we chose the AmpliSeq chemistry and panel (Ion AmpliSeq SARS-CoV-2 Research Panel) with IonTorrent sequencing technology for the sequencing of the whole SARS-CoV-2 genomes, with the additional aim to improve the efficiency of the chemistry on low-template samples.

The available literature shows the full-genome sequences can be obtained even from samples with less than 100 viral RNA copy number per reaction by analogous workflows (Ion AmpliSeq SARS-CoV-2 Research Panel and Ion Torrent Genexus Integrated System) [28]. This is just below the lowest number of viral RNA copies for which we have obtained high-quality results (156 viral RNA copies per reaction, sample 365; see Table S5). Consistent with our study are findings by Jacot et al. [29] who suggest C_T_ ≥ 30 (78 or less of viral RNA copy number per reaction) can serve as an initial sequencing success predictor. An analysis comparing the Ion AmpliSeq Panel and Illumina-MiSeq ARCTIC Protocol showed both methods are overall equally effective, with the automation of all steps of library preparation within the TFS solution considered a strong advantage [30], which was expressed also by Rachiglio et al. [28]. In the beforementioned study [30], one low-quality sample (C_T_ 32,5) achieved 89% coverage with MiSeq-based ARCTIC pipeline, while it failed with the SARS-CoV-2 AmpliSeq Research panel, suggesting the TFS solution may be inferior to the MiSeq-based one in cases of low-template material. The reason for this may be however that target region amplification for all samples with the TFS protocol was performed in [30] for only 17 cycles, while in our study, samples with C_T_ over 27 were amplified for 27 cycles, as per the manual, and for a number of samples results were still obtained (Table S5).

Since according to the user manual all steps of library preparation are performed on the basis of the initial assessment of viral RNA copy number within the material, a potential bias introduced in the quantification itself can play a major role in results of the whole analysis. The source of the bias could be the stochastic effect, occurring in extremely high- or low-template samples, as well as the existence of additional targets for primes and probes included in the RT-PCR kits. Consequently, part of the discrepancies of viral RNA copy number levels quantified by different real-time RT-PCR tests [31], and subsequently differences between expected and actual library quantity, can be explained by the presence of subgenomic RNA potentially detected by the SARS-CoV-2-detecting kits. Nonetheless, the existence of those targets can also be beneficial—even though real-time PCR methods targeting SARS-CoV-2 genes cannot differentiate between an active infection and early convalescence, subgenomic RNA-based solutions have been created [31,32,33] and successfully implemented in clinical studies [34,35,36]. Furthermore, Williams et al. [37] suggest viral load may be variant dependent, with the B.1.617.2 showing a 21-fold increase in viral copy number compared to the other variants, thus showing another variable that could potentially be added to the list of factors influencing the library preparation success.

The superiority of the no-normalization protocol over the normalization protocol was clearly demonstrated by the percentage of samples that exceeded the optimal library quantity value within each group, as well as the minimal number of viral RNA copy within a sample to meet the library quantity condition. This could be caused by a stochastic effect occurring in quantification of extremely high or extremely low quantities of viral RNA, additional freeze–thaws or sample handling that could lead to sample degradation, or possible human errors introduced in various multi-step dilutions of samples. Alessandrini et al. [38] evaluated the Ion AmpliSeq SARS-CoV-2 Research Panel in the very beginning of the pandemics (article submittance: July 2020) on the basis of 13 libraries constructed within a study on 10 SARS-CoV-2-positive patients, from whom nasopharyngeal swabs were taken for either direct RNA isolation or cell culture infection followed by isolation of RNA from the pellet. The authors found a negative correlation between number of amplification cycles and uniformity of reads, namely, the 12-cycle amplification protocol resulted in a significantly higher uniformity than the 20-cycle protocol did, leading to the conclusion that with a higher number of PCR cycles, a serious bias caused by differential amplification is introduced. In the study presented here, while the correlation between number of amplification cycles and sequencing quality metrics was not measured (as the number of cycles was adapted to viral RNA copy number within samples), as opposed to the work of Alessandrini et al., we found a positive correlation between uniformity and quantity of library obtained from the sample, as well as with some other sequencing quality metrics (total reads, on target reads). In our case, the protocol that roughly adjusted the number of amplification cycles to the quantity of initial RNA input (no-normalization protocol) resulted in a higher quantity of library that the one that used the exact expected number of viral RNA copies for the target amplification (normalization protocol). This indicates that as some overamplification does not cause the results to be biased, a significant difference between number of cycles suggested for a specific cDNA (or RNA, as in one protocol described in [38]) input and the actual number of PCR cycles used may cause a high variation of amplification rates throughout the panel. In our no-normalization protocol, the highest difference between used and suggested PCR cycle number is five (potentially resulting in 32× more PCR product than advised), while the 20 cycle protocol used by Alessandrini et al. [38] resulted in up to eight excess PCR cycles, which translates to up to 256× more amplicons.

Although for some individual samples the concentration of cDNA helped to reach a higher level of library quantity, overall, it did not show improvement in the results of library construction or sequencing of low-template samples. As the quantity of amplification template was doubled through the concentration, one possible explanation for its failure can be the cDNA concentration factor being too low to impact the results significantly. To further assess the potential of concentrating the samples, more RNA could be reverse-transcribed and then concentrated, or more isolates from one sample could be concentrated and then cDNA could be obtained from the thus-prepared template, both resulting in a higher concentration factor of the initial sample. Still, too high a concentration factor can lead to an elevated level of PCR inhibitors, potentially impacting the robustness of the amplification chemistry; among some known inhibitors that caused the former TFS library kit (HID-Ion AmpliSeq Library Kit) [39] and the current forensic genetics-dedicated one (Precision ID) [40] to fail, is hematin [39], a compound that impairs DNA polymerase activity [41].

Lineage-dependent amplicon failure was one of the aspects studied by Tan et al. [42] in a sample set that contained six isolates obtained from swabs collected between April and May of 2020 in Malaysia. The amplicons that highly underperformed in that study (r1.14.786182 and r1.25388943) were failing for all but one sample, which contained some changes in comparison to the reference Wuhan-Hu-1 sequence nearby the amplicons’ range. This suggested that the mutations in question could potentially improve primer binding, while their alternate variants may have caused the amplicons to underperform. In our dataset, however, those amplicons were not failing (mean percentage of reads within all reads of 0.307% and 0.310%, respectively), even though the 13730T>C and 23929T>C variants did not occur in any of our samples. Moreover, the amplicon that failed consistently throughout our samples did not show underperformance in the Tan et al. study [42]. The possible explanation for this may be that even though there are no indications that the high underperformance of r1_1.15.1421280 is caused by a specific SNP, the collection time of samples studied by Tan et al. pinpoints them to the period when B.6 was the main lineage that prevailed in Malaysia [43]. In comparison, our study set did not contain any sample assigned to the B.6, thus suggesting that the mechanism of failure of some amplicons may be more complex. Alessandrini et al. [38] found a 4255G>T transversion could be the reason for r1_1.5.1289446 underperformance. Our results support that assumption, with the 4255G>T variant found in four samples causing a high underperformance of the r1_1.5.1289446 amplicon in each of those samples; the underperformance itself was not associated with any specific SARS-CoV-2 lineage. Overall, we found a number of SNPs, present within different SARS-CoV-2 lineages, that cause the neighboring amplicons to underperform. The proximity of those SNPs to the amplicon borders strongly suggest that they interfere with primer binding and either disrupt or disadvantage the formation of the amplicon. This places amplicon-approach sequencing among other methods that can be impacted by SNPs located at primer binding sites, such as qPCR [44] and fragment analysis by capillary electrophoresis [45].

Our data showed that in different lineages, up to eight amplicons can underperform, potentially causing some sequence to be lost in various parts of the genome, while the construction of the panel in a way that amplicons overlap reduced the number of bases being lost. Additionally, we observed the presence of gaps within genomes obtained from 53 samples that had to be handled manually, as they were returned by the GISAID. A high frequency of gaps across the supposedly complete genome sequences occurs frequently within the sequences deposited in the GISAID database, which is suspected to be caused by the wide use of amplicon-approach sequencing [46]. As those gaps occur in results obtained through both Illumina- and Ion Torrent-based platforms, their presence is likely linked to the approach itself, rather than a specific technology, and could be due to primer trimming issues during the read data quality control, unpredicted interactions between primers, and/or variability of target regions [46]. On the basis of our results, we recommend all widely used panels to be monitored and updated, analogous to the way RT-PCR diagnostics methods are observed by the FDA [11]. Some alternate primers have been proposed already [46,47]. Another way to address this issue might be the inversion of library preparation protocol. Alessandrini et al. [38] tested if the transcription performed in the same reaction as the target region amplification can improve variant-independent primer binding, and the results were promising. However, the single-step transcription and amplification is also implemented in commercially available RT-PCR kits, which are still prone to underperformance due to genetic changes of the virus, in spite of the combined protocol [11].

## 5. Conclusions

Even though some amplicons of the AmpliSeq™ SARS-CoV-2 Research Panel highly underperform specifically within some lineages, for all of them, we were able to obtain full or near-full sequences. Given the constant evolution of viruses, NGS is a powerful means for viral surveillance; nonetheless, the performance of widely used research panels needs to be monitored closely in the event of the emergence of new strains. Using one kit through time can cause some parts of the genome to be missed consistently, especially with highly mutated variants—for the B.1.1.529, the number of gaps can potentially add up to 132 nucleotides only within the Spike protein gene alone.

Our data show the modified library preparation protocol (no-normalization protocol) produces better results than the one originally proposed by the manufacturer, while concentration of cDNA did not significantly improve the library preparation or sequencing of low-quantity samples.

## Figures and Tables

**Table 1:** Overview of samples subjected to library construction, amplification protocols, and quality of constructed libraries.

Sequencing Batch	Number of Samples	Samples with Optimal Viral Copy Number per Reaction (≥200)	Samples that Yielded Optimal Library Conc. (≥70 pM)	Reamplified Libraries	Reamplified Libraries that Yielded Optimal Conc. (≥18 ng/mL)	Number of Fasta Files Obtained	Amplification Protocol
R1	80	78	65	9	8	80	NORMALIZATION PROTOCOL
R2	80	73	50	30	29	79	
SUBTOTAL ^1^ (R1–R2)	160	151	115	39	37	159	
R3	79	47	54	25	21	79	NO-NORMALIZATION PROTOCOL
R4	79	78	78	1	0	78	
R5	77	51	75	2	0	77	
R6	77	76	77	0	-	77	
R7	81	81	81	0	-	81	
R8	81	81	81	0	-	81	
SUBTOTAL ^2^ (R3–R8)	474	414	446	28	21	473	
TOTAL	634	565	561	67	58	632	

^1^ normalization protocol: samples were divided into three groups on the basis of the C_T_ value: ≤22 samples were normalized to 20,000 viral copy number/reaction and amplified for 17 cycles; 23–25 samples were normalized to 2500 viral copy number/reaction and amplified for 20 cycles; 26–31 samples were normalized to 78 viral copy number/reaction and amplified for 25 cycles; ^2^ no-normalization protocol: samples were divided into four groups on the basis of the C_T_ value: 12–17 samples were amplified for 13 cycles; 18–22 samples were amplified for 18 cycles, 23–27 samples were amplified for 23 cycles; >27 samples were concentrated for 15 min in 45 °C, afterwards water was added for a final volume of 10 μL, and samples were amplified for 27 cycles (conc.–concentration).

**Table 2:** Results of concentration of low-template RNA isolates (conc.–concentration).

		N GENE CT	VIRAL RNA COPY PER REACTION	LIBRARY CONC. (pM) (TARGET 70 pM)	REAMPLI-FIED LIBRARY CONC. (ng/mL) (TARGET 18 ng/mL)	READS	ON TARGET	MEAN DEPTH	UNIFORMITY
LOW-TEMPLATE SAMPLES SUBJECTED TO RNA CONCENTRATION	MEAN	30.1	99.3	340.1	73.0	525,759.0	72.06%	2380.7	42.96%
	MEDIAN	29.0	78.0	86.6	31.6	347,048.0	84.94%	503.9	38.67%
	SD	2.0	50.9	721.1	106.3	560,685.2	26.48%	3126.2	30.29%
LOW-TEMPLATE SAMPLES, NOT SUBJECTED TO RNA CONCENTRATION	MEAN	29.0	104.0	100.2	105.9	566,962.3	74.48%	2174.1	43.41%
	MEDIAN	29.0	78.0	74.7	91.4	580,470.0	77.98%	718.1	38.91%
	SD	1.1	48.6	90.3	68.5	440,854.7	16.89%	2370.6	26.86%

**Table 3:** Mean parameters of samples and libraries constructed within the study; basic quality metrics of sequencing runs (conc.–concentration).

		N GENE C_T_	VIRAL RNA COPY PER REACTION	LIBRARY CONC. (pM) (TARGET 70 pM)	REAMPLIFIED LIBRARY CONC. (ng/mL) (TARGET 18 ng/mL)	READS	ON TARGET	MEAN DEPTH	UNIFORMITY
SUMMARIZED	OVERALL	20.7	517,043.1	1432.7	72.0	1,096,133.6	94.74%	6866.4	80.66%
	R1–R2	19.5	103,894.7	283.6	72.4	1,053,337.7	96.28%	6400.0	73.60%
	R3–R8	21.8	685,312.1	1817.4	71.5	1,110,459.0	93.97%	7023.5	84.18%
OPTIMAL CONC.	OVERALL	20.4	578,483.9	1616.2	-	1,209,977.6	97.18%	7618.0	88.93%
	R1–R2	18.7	122,172.9	379.4	-	1,348,146.2	97.64%	8315.9	85.08%
	R3–R8	21.5	713,803.7	1919.5	-	1,176,098.3	97.01%	7446.2	90.34%
OPTIMAL INITIAL RNA INPUT	OVERALL	19.7	574,396.9	1570.4	66.3	1,165,060.8	98.38%	7411.8	86.80%
	R1–R2	19.0	108,337.3	292.7	66.3	1,078,694.5	97.28%	6639.7	74.95%
	R3–R8	20.3	775,134.2	2036.4	-	1,196,561.5	99.02%	7694.9	93.68%
OPTIMAL CONC. AND INITIAL RNA INPUT	OVERALL	20.1	737,264.0	1772.5	-	1,213,934.0	98.71%	7750.6	91.40%
	R1–R2	19.5	124,233.1	398.5	-	1,311,516.7	97.72%	8094.1	84.08%
	R3–R8	20.3	924,833.2	2044.0	-	1,194,652.6	99.03%	7682.4	93.70%
REAMPLIFIED	OVERALL	23.1	33,212.4	29.4	72.0	167,032.7	80.00%	723.8	32.84%
	R1–R2	22.3	45,257.7	30.9	72.1	248,339.7	90.58%	1142.5	37.93%
	R3–R8	31.0	87.8	27.4	72.0	53,783.7	64.71%	119.1	25.48%
REAMPLIFIED WITH OPTIMAL CONC.	OVERALL	22.6	35,579.1	29.5	81.5	184,595.0	79.81%	801.0	35.36%
	R1–R2	22.3	45,257.7	30.9	72.1	248,339.7	90.58%	1142.5	37.93%
	R3–R8	33.0	91.0	26.5	100.9	53,750.7	57.71%	100.0	30.07%

**Table 4:** Amplicons with low mean percentage of reads within total sample’s reads for specific SARS-CoV-2 lineages and potential SNPs associated with amplification failure.

LINEAGE	NO OF SAMPLES	TARGET REGION	RANGE		RANGE LOST IF AMPLICON FAILS		MEAN % READS WITHIN ALL SAMPLES	CHANGE	POSITION OF VARIANT IN RELATION TO AMPLICON RANGE ^1^	VARIANT	% READS
**B.1.1.306**	**N = 1**	**r1_1.1.592753**	**316**	**535**	**484**	**512**	**0.854%**	**313C>T**	3U		
B.1.1.44	N = 1	r1_1.10.67083	9179	9390	9199	9251	0.199%	9166C>A	13U	**T**	0.005%
B.1.389	N = 1	r1_1.10.711902	9432	9643	9477	9561	0.377%	9430C>T	2U	T	0.004%
										T	0.002%
B.1.1.317	N = 1	r1_1.11.376074	9857	10,071	9906	9952	0.388%	9857C>T	0U	T	0.008%
AY.9	N = 2	r1_1.12.1117806	11,523	11,730	11,634	11,684	0.344%	11514C>T	9U	T	0.002%
B.1.160	N = 2	r1_1.12.539895	11,260	11,477	11,366	11,410	0.434%	11497C>T	20D	T	0.082%
B.1.1.306	N = 1	r1_1.13.620498	12,563	12,790	12,711	12,772	0.630%	12805T>C	15D		
AY.1	N = 2	r1_1.15.1421280	14,410	14,550	14,473	14,514	0.007%	14408C>T	2U		
AY.9	N = 2										
B.1	N = 9										
B.1.1	N = 22										
B.1.389	N = 1	r1_1.16.1212393	15,560	15,741	15,583	15,624	0.301%	15543G>T	17U		
B.1.258	N = 12	r1_1.16.534874	15,387	15,582	15,522	15,559	0.879%	15598G>A	16D		
B.1.389	N = 1	r1_1.21.272458	20,380	20,604	20,457	20,497	0.372%	20622A>T 20623G>T 20624A>T	18D		
B.1.1.398	N = 4	r1_1.22.1029456	21,187	21,255	21,199	21,245	0.409%	21178C>T	9U		
B.1.2	N = 5	r1_1.22.906171	21,309	21,531	21,346	21,450	0.351%	21304C>T	5U	**A**	0.002%
										**A**	0.004%
AY.9	N = 2	r1_1.23.1186794	22,246	22,446	22,320	22,367	0.407%	22227C>T	19U	T	0.006%
B.1.177	N = 9									T	0.004%
B.1.177.8	N = 1									T	0.003%
										T	0.009%
										T	0.006%
AY.1	N = 2	r1_1.23.127614	21,757	21,973	21,803	21,846	0.095%	21987G>A	14D		
AY.9	N = 2										
AY.1	N = 2	r1_1.23.86525	21,847	22,058	21,974	22,024	0.325%	21846C>T	1U		
B.1.617.2	N = 1										
B.1.389	N = 1	r1_1.24.394902	23,580	23,724	23,643	23,682	0.601%	23730C>T	6D		
AY.9	N = 2	r1_1.24.626090	23,008	23,224	23,049	23,088	0.380%	22995C>A	13U	A	0.022%
AY.1	N = 2										
B.1.221	N = 32	r1_1.26.1209362	25,666	25,887	25,797	25,844	0.249%	25906G>C	19D	**T**	0.008%
										C/G (1382/3601)	0.284%
B.1.1.121	N = 3	r1_1.27.410513	26,651	26,847	26,801	26,838	0.424%	26645C>T	6U		
AY.9	N = 2	r1_1.3.760885	2398	2616	2300	2463	0.263%	2388C>T	10U		
B.1.258	N = 12	r1_1.30.1041188	29,498	29,727	29,530	29,592	0.328%	29734G>C	7D		
AY.1	N = 2							29742G>T	15D	T	0.013%
AY.9	N = 2									T	0.029%
										T	0.100%
B.1.1.159	N = 2	r1_1.5.75163	3891	4110	3973	4018	0.240%	4114T>C	4D		
B.1.1.306	N = 1							3879–3899del	12U		
B.1.1.44	N = 1	r1_1.6.1402513	4920	5129	5028	5073	0.385%	5147C>T	18D	T	0.024%
AY.9	N = 2	r1_1.6.888565	5587	5810	5671	5716	0.481%	5584A>G	3U		
B.1.1.317	N = 1	r1_1.7.488632	6293	6512	6339	6379	0.561%	6536G>A	24D		
B.1.177	N = 9							6286C>T	7U		
B.1.177.8	N = 1										
B.1.1.44	N = 1	r1_1.8.592180	6804	7017	6830	6882	0.210%	-			
B.1.1.159	N = 2	r1_1.8.816048	7149	7360	7284	7331	0.149%	7379G>A	19D		

^1^ U—upstream, D—downstream. Underperforming amplicons are marked red. Variants that differ to those found in lineages are bolded and underlined. % READS—percentage of amplicon’s reads within total reads for the sample that shared the variant.

**Table 5:** Prediction of amplicons with low mean percentage of reads within total sample’s reads for SARS-CoV-2 VOC B.1.1.529 and potential SNPs associated with amplification failure.

LINEAGE	NO OF SAMPLES	TARGET REGION	RANGE		RANGE LOST IF AMPLICON FAILS		MEAN % READS WITHIN ALL SAMPLES	CHANGE	POSITION OF VARIANT IN RELATION TO AMPLICON RANGE ^1^
B.1.1.529	N = 5	r1_1.11.528369	10,453	10,679	10,603	10,655	0.197%	10449C>A	4U
		r1_1.15.1421280	14,410	14,550	14,473	14,514	0.007%	14408C>T	2U
		r1_1.23.127614	21,757	21,973	21,803	21,846	0.095%	21987G>A	14D
		r1_1.23.474025	22,494	22,685	22,582	22,622	0.436%	22686C>T	1D
		r1_1.24.942468	23,089	23,290	23,225	23,268	0.256%	23075T>C	14U
		r1_1.26.781963	25,372	25,566	25,478	25,511	0.785%	25584C>T	18D
		r1_1.27.993816	26,588	26,800	26,606	26,650	0.332%	26577C>G	11U
		r1_1.29.497787	28,374	28,606	28,463	28,512	0.521%	28,362–28,370del *	4U

^1^ U—upstream, D—downstream. Underperforming amplicons are marked red. * Deletion present in four out of five samples tested.

## Data Availability

The sequences used for lineage assessment are deposited in the GISAID under accession numbers: EPI_ISL_10301369-377, EPI_ISL_10301379-519, EPI_ISL_10301521-602, EPI_ISL_10301603-719, EPI_ISL_10301721-727, EPI_ISL_11068565-EPI_ISL_11068614, and EPI_ISL_11069509-EPI_ISL_11069511.

## Supplementary Materials

The following supporting information can be downloaded at https://www.mdpi.com/article/10.3390/v14061230/s1, Figure S1: title; Table S1: title; Video S1: title. Table S1: Alignment quality within each sequencing run. Table S2: Correlation calculations. Table S3a: Amplicon success rates (sorted by mean percentage of amplicon’s reads within total reads, highest to lowest). Table S3b: Amplicon success rates (sorted by percent of samples with <50 reads, lowest to highest). Text S1: Sequences of South African samples chosen for B.1.1.529 amplicon failure prediction not available in the GISAID. Table S4: Sample, library, and sequencing metrics. Table S5: Sequencing quality metrics within different sample and library groups.
